# Dissertation on the Claims of Medical Science upon the Practitioner of Dental Surgery

**Published:** 1844-09

**Authors:** Amos Westcott


THE AMERICAN

### JOURNAL OF DENTAL SCIENCE.

Vol. V.] SEPTEMBER, 1844. [No. 1.

### ARTICLE I.

*Dissertation on the Claims of Medical Science upon the Practitioner of Dental Surgery.* Read before the American Society of Dental Surgeons, at its Fifth Annual Meeting, held in the City of New York, July 17th, 1844. By Amos Westcott, M. D., D. D. S.

In discharging the duty assigned me by this society, at its last annual meeting, I propose to present some of the many claims of medical science, *as preparatory to the pursuit* of dental surgery. When I reflect upon the importance of these claims, I could wish for them an abler advocate—one whose age and station would tend to give sanction to his sentiments. The stand which dental surgery is taking among the learned professions, and that far from limited range of diseases, which are now justly considered as coming within the province of the dentist, requires of him something more than those qualifications, fitting him for a mere *tooth-smith.* The public are beginning to require at his hand, the exercise of duties which science alone will enable him properly to discharge.

These claims can, perhaps in no way, be more clearly presented, than by an examination of its several branches. Before entering upon detail, however, we shall premise, that he who practices every branch of dental surgery safely, must be sufficiently acquainted with the human system, not only to trace

13373

given *symptoms* to their *cause*, but able to anticipate all their possible tendencies; for, as it has been justly observed, while his profession would seem to limit him to one suit of organs, these are "organs connected most intimately, by a thousand sympathies, with almost every part of the system."

He would be esteemed a poor general, who should know only the situation of the battle ground, while he was ignorant of all the thousand avenues to and from it, by which the enemy might retreat, or be reinforced; and it is equally true, that he who undertakes to meet, and repel disease, the common enemy of health and life, should not only be familiar with its present aspect, the ground which it at the time occupies, but with all its connections, with other parts.

To do this in respect to the human system, a knowledge of *anatomy*, which alone can give us definite information of the location, and structure of every organ, and their connections, and dependencies, is absolutety essential.

Sir Charles Bell declares it to be the basis of all medical skill, and all will doubtless concur in this opinion, who are sufficiently acquainted with it, justly to appreciate its bearings upon *any* branch of the healing art. Its claims upon the dentist are manifold. It is important as the only means of obtaining a clear idea of those parts directly involved in dental operations—to give him the relative connection and dependencies of these parts with each and every other part of the system, affected through the medium of the nerves, circulation, and its tributaries, respiration and digestion—and as being the basis of several other branches of medical science.

In proceeding to illustrate these several claims, we remark that the necessity of understanding those parts directly concerned in dental operations, is so nearly self-evident, that it hardly needs illustration. Few, and did not observation *prove* otherwise, we should say none, calling themselves dentists, are so culpably ignorant, as not to understand the anatomy of the teeth, their general form, both within and without the jaw; the number and relative position of the fangs of each class; the structure of the maxillary bones, and in short, with each part *directly* involved in his operations; but the idea of studying

*remote* organs, while his operations seem confined to the mouth, is to the mere *head*-anatomist, a stumbling-block, and to the mechanical dentist, foolishness. But we have only to extend our observation a little, and examine the multifarious connections between different organs, to be clearly convinced, that no organ of the body can be regarded or intelligibly studied as an independent integer. The functions of the human system, the harmonious action of which, constituting health and life, have been properly compared to a circle—beginning where we may, we find each intimately connected with another part, essential to the whole, and in whatever direction we pursue our examination, we ultimately arrive at the starting point. If, for instance, we commence our investigation upon the *brain*, we find this dependant upon the heart and lungs for its supply of arterial blood—if upon the *heart*, this is dependent upon the brain for nervous energy, and upon the lungs to purify its blood,—and if upon the *lungs*, we find that their action cannot be sustained, without both the influence of the nervous system, and the action of the heart, to propel through them the vital fluid necessary for the support of the whole. Again, each of these are dependant on the stomach, for its appropriate nourishment, while the stomach, in turn, requires their combined agency in order to perform properly its function.

But, on the present occasion, we shall confine our remarks principally to the connection of organs by the *nerves*—those delicate strings of the grand instrument, the human body, whose harmonious vibrations communicate health and vivacity throughout the system, but whose discordant strains are heard in the shriek of distress, or the agonizing groan of the dying.

We shall also, briefly notice the connection of parts, through the medium of the circulating fluid, carrying with it either health or disease to be imparted to every organ. Without giving a detailed description of the particular classes of nerves, it may be premised, that they are connecting cords, passing from the grand sensorium to each organ of the body, imparting to it, either action or sensation, and upon which, the organ is dependant for the appropriate exercise of its function—and as branches of the nerves, supplying particular organs, freely unite with those supplying different ones, they serve also, to secure that simultaneous and harmonious action of the various parts of the system, upon which life necessarily depends. It may, indeed, be assumed, that *each nerve is connected with every other nerve, in the entire system.* The whole system of organs is thus connected, as is the solar by gravity, which keeps each planet revolving in its own proper sphere. To balance a single planet, affected only by its primary, so that it would travel in a regular orbit, would be an easy problem; but when we reflect that not only every *planet*, but every *atom*, in the universe, is affected by every other, however minute or remote, and then find that all move on in harmonious concert, we can but see and admire the wonder-working of an Almighty hand. We see, moreover, that if the least of the ten-thousand, were to be thrown from its true orbit, confusion must reign throughout the whole.

If possible, even greater skill seems to have been expended in the construction of the surprisingly curious machinery of the nervous system. While particular nerves are destined to supply particular parts, they freely anastomose with those sent to other organs, and receive, in turn, filaments from them, thus perfectly uniting every organ of the body into one entire whole—and as in the solar system, should one part be thrown from its balance, it would disturb the equilibrium of all the rest, so in the human system, when any one organ is affected by disease, the whole, to a greater or less extent, must partake of the derangement.

Dr. James Johnson, in speaking of the sympathetic connection established between the different organs, through the agency of the nervous system, thus remarks:

"The brain and its appendages, being the medium through which the living principle acts upon the otherwise inorganic structure, as also the medium through which the *mind* is made conscious of injurious applications offered to any part of the machine, it may be properly said, that *every part* of the human body is capable of sympathizing with the rest, because we see that *no* part can admit of having the sense of feeling excited to the height of pain, without the *whole* frame being deranged in sensibility."*

* This no one could fail to anticipate after studying the functions and connections of the different nerves.

Although it would be foreign to our present object, to describe, in detail, *all* the different nerves of the human body, and their manifold connections, yet we may, with propriety, allude to some of the particular nerves, and notice their important connections. As an example, let us notice the fifth pair. This pair of nerves, after serving its office in giving "sensation to *every part of the head and face*, and appendages, and, as supposed by Charles Bell, special sensation to the tongue, communicates with the *third*, the *sixth*, *seventh*, the *eleventh*, and with the *great sympathetic*, forming of itself a kind of sympathetic nerve, by which not only all parts of the *head* are connected with each other, but *all other parts* of the body."

Again, turning our attention to but one of the three branches composing the eighth pair, the par vagum, we find it distributed to no less than twelve separate organs!—"the œsophagus, pharynx and larynx—to the thyroid gland—to the vessels of the neck and heart, to the lungs, liver and spleen, stomach and duodenum, and to the diaphragm."

After showing this connection of important parts, affected by this single nerve, it would seem superfluous to point out the healthy and morbid influences which would be thus established between these organs. The recollection of this description, says Mr. Bell, will explain to us many sympathies—"for example, the irritability of the larnyx in exciting coughing, the hysterical affection of the throat, when the stomach is distended with flatus, the exciting of vomiting by tickling the throat, the effect which vomiting has in diminishing the sense of suffocation, the relation between the heart and lungs, the lungs and stomach, and the stomach and heart."

A very similar description in respect to the union of different parts, and organs, may be given of all the nerves arising from the brain, except those of special sensation, and to a very great extent, to those of the spinal marrow.

But beside the unity of action established by the almost endless interchange of filaments of encephalic nerves, one more perfect, if possible, is formed by the ganglionic system, or the grand sympathetic nerve; which Mr. Bell describes to be "a truck of medullary matter passing through, and connecting, the head and neck, viscera of the thorax and abdomen, and pelvis, into one whole—and the extent of its connections occasions, both in health and disease, sympathetic affections, not easy to trace."

Viewing as we are constrained to, even by this imperfect glance at offices and connections of the nerves, the human body, as composed of many parts united into one, we inquire what organ or part can be thoroughly understood without some knowledge of the whole? As well might we expect to obtain a correct and thorough knowledge of a clock, by an examination of *one* of its wheels.

The most skilful *physician* is he who best understands these connections, and is, hence, best enabled to trace, or, indeed, *anticipate* the morbid sympathies which are likely to be induced by any given condition of a particular organ. It is said of Dr. Darwin, that there was scarcely a town on the continent of Europe from which he was not consulted, either personally or by letter, and we have it from the same good authority,* that his success was mainly owing to the multiplied resources which the sympathetic view of disease opened out to him.

We are now prepared to appreciate some of the beauties and perfections of "head-anatomy." I will admit, says one, that the dentist ought to study the anatomy of the head! But let us see how wise one would become by confining his researches to the head. And here, permit me to inquire, what good reason can be assigned for studying the anatomy of the *head*, in distinction from *general anatomy*? Surgery recognizes no law of contiguity, or even contact, unless between like tissues, or structures. If we examine every disease coming under the general class of *phlegmasiæ*, no fact will be more apparent, than that inflammation is governed by other and different laws. For example, in *phrenitis*, both the duramater and piamater are inflamed, without materially affecting the arachnoid membrane, although in immediate contact. Again, in arachnitis, the delicate arachnoid membrane becomes inflamed, without imparting its effects on the one hand to the piamater, or, on the other, to the duramater, nothwithstanding these adhere to it on either side! In *pleuritis*,

*Dr. James Johnson.

also, we see a delicate membrane passing through the several stages of inflammation without involving the contiguous structures. Though an aphthous sore-mouth may extend to the throat, and even the entire length of the alimentary canal, by virtue of similarity of tissue, yet we never hear of its producing a similar effect upon the face, nor of its giving rise either to ophthalmia or tenea capitis. We might multiply these illustrations till we had enumerated every organ and tissue of the entire system. Considering it, however, settled, that the law of contiguity does not prevail in determining the extension of disease, we again ask, why study the anatomy of the entire head? This, it is true, would include the parts immediately concerned in dental operations, in case the student, by such limited reading, is capable of understanding anatomical description, and this is certainly very well—but why not confine the attention to these *necessary parts*, if the other portions of the head are no more in jeopardy from a diseased denture, than remote organs? Does he reply that the teeth have nerves, and that they must be studied? Let us for a moment turn our attention to these *dental nerves*, and inquire what proportion of the nerves of the head pertain directly to the teeth. Of the eight pairs which have their origin in the brain, only a *single twig*, from two of the branches of a *single pair*, goes to supply the teeth. This (the fifth pair) is divided into three great branches—1st, ophthalmic, 2d, the superior maxillary, and 3d, inferior maxillary. The first, or ophthalmic, with all its six branches, goes entirely to other parts than the teeth.

The second, or superior maxillary, gives one, of seven branches, to the teeth and sockets of the upper jaw—and

The third, or inferior maxillary, gives only one, of nine branches to the teeth and sockets of the lower jaw.

We are now prepared on this exclusive system, to assign to the dental student his exact task in *dental anatomy*. He is to study the teeth, the parts in immediate connection, and *one-eleventh* of a single pair of nerves! Should he reply that these nerves should be farther studied on account of their connections, he is referred to our foregoing remarks upon the union of these nerves—that while they are connected with others supplying the head, they are also as intimately connected with those supplying remote organs. Let those, therefore, who would spurn the idea of the dentist studying general anatomy, never condemn a few weeks' studentship in dentistry, nor wonder that a "single manufactory of dental blockheads should send abroad from eight to twelve per annum" of students, when their course is so extremely limited. We believe that this time is not at all disproportionate to the task, and we think, moreover, that for all the tuition called for, in ordinary cases, "a barrel of molasses" *would be* a generous compensation. Surely this is no mean reward, for so trifling a service—it would serve to sweeten the cares of many a wearisome day.

It is often urged that those diseases, although connected with the teeth, which require a general knowledge of the system, do not properly belong to the dentist to treat. This transfer of patients might be admissible, did the limited knowledge requiring such transfer enable the dentist *when* it should be made—it being a fact, that it often requires much more skill to recognize the true nature of a disease, than to treat it successfully after its real character is understood. But even were this practicable, to remain in ignorance of all those things which *may* be passed into the hands of another, betrays a culpable stupidity, excusable only in those animals, remarkable less for their volume of brain than length of ears. In this way every thing connected with dentistry might be transferred either into the hands of the physician, surgeon or mechanic, leaving himself, what he ought to be regarded by the public, a mere cypher, worse than an apology for a man.

This cursory view of the claims of anatomy upon the dental student, although from the limited nature of this essay, necessarily imperfect, is, nevertheless, sufficient to show the importance of the study of *general anatomy*, to all who practice *any* department of the healing art. Our argument has, thus far, been founded upon an examination of structure, which would only enable us to predict sympathetic disease, from the connections shown to exist; but our proof is far from stopping here. The history of disease is but the record of this almost universal transfer of morbid action through the medium of sympathy. We might here proceed to give examples in illustration of this position, but these will more properly come under our remarks on the practical branches of medicine. Not only is disease thus transferred from organ to organ, and often to those very remote, but the action of many *remedies* are to be understood only by a knowledge of these connections. This principle is, indeed, *solely* relied upon in all cases where the remedy is not directly applied to the part affected, which is true only of external local applications. But in all cases of the administration of general remedies, the affected organ or part, is reached only through the agency of the circulation, or nervous communication.

In our investigation of the human system, after the study of the structure of the different parts of the body, very naturally follows the inquiry relative to the office or function of the various organs, or *human physiology*, whose claims upon the attention of the dentist, we shall next consider. Under this head, though there are many subjects that might be profitably introduced, we shall confine our remarks on the present occasion, principally to the *circulation of the blood*, and the tributary functions of *respiration* and *digestion.* These subjects are fraught with interest, not only as presenting some of the most important physiological phenomena, but they are of peculiar interest to the dental surgeon. While their careful investigation will present to him facts which will be of the greatest practical utility, he can but clearly see in them the correctness of the position we took in the beginning of our remarks—that no organ can be properly studied, isolated from the rest.

But for a regenerating process constantly going on, the system would soon go to waste. The victory in the perpetual warfare between vitality and affinity would soon be gained by the latter—the fairest form would lose its comeliness, "the sparkling eye its brilliancy, and beauty its fascinating charms." This regeneration is effected by the *blood*, in its circulation through the system. While this vitalizing fluid travels to every organ and part, loaded with renovating material, it would also soon become exhausted, but for its constant accessions through the agency of respiration and digestion The nutritious portion of the food taken into the stomach, after the process of digestion is completed, in the form of chyle, is, by the lacteals, carried to the left subclavian vane, thence to the right side of the heart. Here it is met by, and unites with, the venous blood, and both pass into the lungs, there to undergo an important change.

Upon the venous blood brought from all parts of the system, the lungs perform a double office. Here by expiration the effete matter, which it has taken up from every part of the system, is thrown off, and from the air inhaled, oxygen is absorbed, changing it from venous to arterial blood—while the chyle brought from the stomach is by the same act of inspiration transformed also, into arterial blood, and both pass on to the left side of the heart, thence to every part of the system. We are now prepared to appreciate the influence of a diseased state of the mouth upon even remote parts, through these two avenues, the stomach and lungs.

It has long been a maxim, that food well masticated, is half digested, and the connection just alluded to, between a healthy and perfect digestion, and the proper supply of nutrition to each organ must be apparent.

Again, from a diseased state of the mouth there is frequently more or less pus and other foul matter swallowed in connection with the food, to say nothing of the ill effects of the poisonous drugs so often resorted to, with the view to allay the pain of an aching tooth, or the constant tax upon the system, by a perpetual local irritation. Nor are these pernicious effects confined to the stomach, but these substances are taken into the circulation, and their influence thus imparted to every organ of the system.

Secondly, a diseased denture imparts its baneful influence to the general system, still more frequently and efficiently, through the medium of the *lungs.*

This will be apparent by a simple statement of a few physiological facts. About eight per cent. of all the air inhaled, is absorbed by the blood in the lungs, and is carried throughout every part of the animal body, acting not only upon the elements of the food, but imparting its effects to every part supplied with arterial blood—yielding, according to its quality, a stimulus to healthy or morbid action, and regulating also the temperature of the whole frame.

The blood of the entire system, (about twenty-five pounds,) is thus subjected to this important change every two and a half or three minutes during life. A knowledge of these facts would lead us to *predict*, *a priori*, that to sustain health a pure air would be necessary. But upon this subject, we are by no means left to conjecture. That to preserve health, a pure air is *absolutely essential*, is a fact that has been fully recognized from time immemorial, and by all classes of society—and, it may be added, that, perhaps, in no department has modern science effected more good to the human family than by pointing out this prolific source of disease, and the most efficient means of removing it.

Although these efforts have done much towards ameliorating the condition of our race, they have hitherto been far too exclusively confined to those sources of miasmi, wholly extrinsic in their origin, to the persons affected by them. In *this* channel of investigation, however, no step has been left untaken, or stone unturned, which could promise additional success. Every suspected district has been examined, and its exhalations carefully analyzed, from those which produce the deadly plague, or those which give rise to the dreadful catalogue of febrile epidemics to those whose effects are witnessed in a form less terrible, though more protracted, the never-ending ague and fever. Nor have these investigations been confined to infected districts of country, but the pestiferous air, generated about the *persons* of the *sick* and the *dying*, and the *dead*, have been equally a subject of scrutiny. In this department, modern science has been efficient in developing the laws governing the formation of these different effluvia, their relation to the human system, the best method of preventing their accumulation, or removing their cause, and the means, in many instances, of neutralizing them when their source could not be removed. But with all the talent and well directed zeal expended upon this subject, it appears to me that one important, prolific, and by no means unfrequent source has been almost wholly overlooked, and, of course, its consequences upon the system.

In vain may one hope to secure to himself the benefits of pure air, either from a salubrious climate, change of place, free ventilation of rooms, or by disinfecting agents, while he carries the very hot bed of infection in the mouth, the baneful influence to be imparted to the system at every breath. Of the fact, that the breath is not unfrequently loaded with putrid matter, I need offer no proof to any practical dentist, and that this air, surcharged with infection, does exert a pernicious influence upon the delicate structure of the lungs, and through the agency of the circulation, on every part of the system, cannot be doubted.

How else does it so often happen, that persons after having sought every aid, which either change of climate, celebrated springs, or eminent medical skill could offer—in short, have tried every expedient which anxiety could invent, or wealth secure, without benefit, have finally had their difficulties entirely eradicated with the removal of their dead and diseased teeth?

The next branch of medical science, whose claims upon the attention of the dentist, we shall consider, is *chemistry.* This science, from the vastness of its resources, has bestowed its benefits upon every department of industry, and upon every other science. It may strictly be said, that there is no science, however elevated, or art, however humble, which is not indebted to chemistry for some of its perfections. But it is foreign to our present object to trace these beneficial results of chemistry in all the various departments in which it has been an active agent, but to inquire what service it is capable of offering to the dental surgeon.

In pursuing this inquiry we will first notice the *indirect* benefits to the dentist, or its applications to medicine. These may be considered under the three following heads, viz.

1st. The light thrown upon physiology.

2d. As the basis of materia medica, including a knowledge of incompatible substances, and the detection and counteracting of poisons.

3d. The remedial agents it points out by an analysis of unhealthy secretions.

In relation to chemistry as a preliminary to physiology, it may be remarked, that many of the subjects discussed in the latter cannot be fully understood without the aid of the former. The phenomena of respiration, calorification, and, to some extent, digestion, may be cited as examples.

It must be observed, however, that when chemical attraction has for an opponent, or even modifier, energetic vital action, we should be slow to adopt any explanation of physiological phenomena based solely upon the laws of affinity. The gastric juice, for example, which readily dissolves *dead* animal matter, and even bone, might be supposed to act detrimentally upon the delicate coats of the stomach, but contrary to any calculation, based on chemical laws, its effects upon the healthy stomach are wholly counteracted by the resisting agency of vitality.

To appreciate the value of chemistry, as the basis of materia medica, we need only compare the present suit of remedies, with those which composed the catalogue less than half a century ago. Instead of using the different articles possessing medicinal properties as furnished by nature, often obliging the patient to swallow a crude mass, a large portion of which was, frequently, not merely inert, but positively noxious, chemistry has furnished almost an entire new catalogue, possessing more concentrated medicinal properties, and freed from the gross and offensive portions which they possess in their native state. Add to these advantages those derived from a knowledge of incompatible substances, the control it gives over hurtful or poisonous articles, even after they are taken into the stomach, and the remedial agents often pointed out by an analysis of unhealthy secretions, and we have some of the many inducements offered to the *medical* student to pursue the science of chemistry. Now, may it not be safely asserted, that the claims of chemistry upon the general student in medicine, are in very few, if in any instances lessened, as applied to the dentist.

In which of the applications of chemistry to general medical science above enumerated, is the dental surgeon uninterested? That he is interested in physiology, and, hence, in all that can shed light upon this branch, we have already endeavored to prove, and that it is true, also, as applied to materia medica, will be evident, when we reflect that the dentist is not only frequently called upon to administer general remedies, but that these are, in a majority of cases, the most potent articles in the whole catalogue of medicines. We go farther still, and affirm, that he is not only *equally* interested with the physician in the application of chemistry to general medical science, but that there are many applications of this science to *dentistry*, in which the physician is little or not at all concerned. With the exception of their application to materia medica, chemical theories are, in most instances as applied to medicine, of doubtful utility, by reason of the modifying agency of the living principle. Although digestion, calorification, and many other functions have been explained on chemical principles, yet in each of these the phenomena are so modified by vitality, that most chemical theories have either been exploded, or substituted by others little less hypothetical.

Not so as applied to *dentistry*. This is the only department of the healing art in which the organs are subject to the full force of chemical affinity—not counteracted, nor even modified by vitality.

In an essay which I had the honor to read before this society, at its last annual meeting, the susceptibility of the teeth to chemical action, was discussed, and the fact that they are liable to be acted upon by any substance, having a superior attraction for one of their constituents, was clearly shown. We have then, on the one hand, the *teeth*, with a definite chemical constitution, and on the other, an extensive list of articles liable to be brought in contact with them, whose affinities are also unalterably fixed. Now to form a correct opinion, as to whether these, or any one of them, as applied to the teeth, will prove harmless, or injurious, either directly or by the fermentation which many of them undergo in the mouth, a most *thorough* knowledge of their relative affinities is *absolutely indispensable.*

Did such knowledge enable us to account for, or prevent only an occasional case of caries, the claims of this science upon the dentist, would be proportionately lessened ; but when we reflect that caries, in *all* cases, is the *direct* result of chemical affinity, of some one or more of these articles, can any one making the slightest pretensions to skill in dental surgery, excuse himself for ignorance of chemistry ? The dentist should not only be able to give to his patients correct information in respect to those substances which are *destructive* in their tendencies, that they may be avoided in all practicable cases, but also the means of counteracting their effects upon the teeth, when they must be used, as in medicine. His duty stops not here. He should be able to analyze correctly, the natural fluids of the mouth, and if these require correction to render them harmless, it is his business to apply the proper remedy. This observation applies to the acid state of the saliva, as also to that condition of it, giving rise to a copious deposition of salivary calculus. I have no doubt that the pathological conditions giving rise to both of these, may be corrected by the administration of proper general remedies. The preparation of dentifrices, and the different local applications for the restoration of the various diseases of the mouth, all of which should be compatible with the chemical composition of the teeth, constitute still further requisitions for knowledge in this science.

Nor is its application confined either to surgical or therapeutic dentistry, but it often renders the greatest service in directing aright, those operations which are regarded purely mechanical. No operation is considered more strictly such, than the insertion of plate teeth. The most common objection to these teeth, especially partial sets, is the injury done to the teeth to which they are clasped. This effect has generally been attributed to the mechanical wearing of these by the clasps—and so long as this error prevails, just so long will this evil lack its appropriate remedy.

That a sound tooth is not capable of being thus acted upon and worn away, by a *gold* clasp, may be shown by direct experiment. Let the tooth be extracted and the clasp made straight, and used as a file, with the view to produce this effect, and what would be the result? Would not a score of such files be destroyed before any considerable impression would be made upon the tooth? A few moments' reflection must convince any one at all acquainted with the relative hardness of the two substances, that such would be the result. What then, constitutes this destructive tendency of clasps?

Chemical knowledge alone, will enable us to solve the problem, or apply the proper remedy. Clasps are seldom, if ever, perfectly adapted to all the irregularities of the teeth, and hence receptacles are formed for the lodgment of food. This, either from spontaneous fermentation or by absorbing, and for a time, retaining substances already acid, or both, rapidly *decomposes* the tooth. The earthy matter being thus taken up, the tooth is, of course, softened, and then, and not till then, the clasp perceptibly wears upon the tooth. We see, thus, that it is chemical, and not mechanical action, that forms the basis of the evil.

The effect would not be materially different, if a simple band of gold, or even *lead*, were to be worn, *unconnected* with a plate. If this view of the subject be correct, it will follow, that any permanent fixture, whether clasp or ligature, is wholly inadmissible, and also that the only safeguard, will consist in perfect cleanliness. Clasps should always, therefore, be so adapted as that they may be easily removed by the person wearing them, and this should be done and both the clasps and teeth thoroughly cleansed immediately after each meal. I venture to say, that that course will effectually blunt the sharp teeth of these *golden files*, and render them quite harmless. In view of the *many* and the *great* advantages which the dentist may derive from this science, permit me to urge the conviction, that his professional study must be exceedingly deficient without a thorough acquaintance, not only with the general principles of chemistry, but with all its important applications to every department of the dental art.

*Materia Medica.*—The primary object of this branch of medical science is to give the natural history, properties, and mode of preparation of those substances used as remedies. Were its application confined to these topics, there could be no good excuse for the dental surgeon to remain in ignorance of this department of medicine. But the term materia medica has a much more extensive signification—it not only includes a description of the natural characteristics of substances, and their sensible properties, but their effect on the living system, the theory of their action, and their application to the treatment of morbid affections; forming so many distinct subjects for investigation. Is the dentist, who is so frequently called upon, not only to apply local but general remedies, to remove some of the most formidable diseases incident to the human frame, to remain in ignorance of the effects of these remedies upon the system? Ought he not, as well as the general surgeon or physician, to have a clear idea of the end to be gained by the administration of any particular remedy, or local application, and the nature and tendency of the article so applied? In short, of which of the topics above named as included in this subject, may the dental surgeon remain in ignorance, and be able properly to discharge the duties of his profession?

The absurdity of such a course is so palpable, that it is idle to waste words to make it apparent. The chief distinction between the quack and man of science is, that the former prescribes for the name of the disease, without reference to its stage, or the accompanying circumstances, whereas the latter makes these the governing conditions in his prescriptions. No fact is more frequently exhibited to the man who thoroughly studies both the nature of disease and remedies, than that in different constitutions the same remedy is frequently not only inadmissible, but is seldom applicable to the different stages of the malady in the same individual. For example, in local inflammatory affections, it may be said, generally, that depletion, either local or general, in the first stage, is the sheet-anchor, but in all of them, in the latter stages, this would be either to jeopardize or to sacrifice the life of the patient; and we may add, that it is only by extensive knowledge, both of disease and remedies, that either the physician or dentist may hope to avoid these dangerous misapplications of remedies.

*General Surgery.*—Various definitions have been framed to distinguish this branch from the general practice of physic, but as a learned professor has justly observed, "the limits between physic and surgery are not very precisely marked, and the respective functions of the physician and surgeon, as long as these names have existed, are still but very inaccurately defined." Surgery having for its object the treatment of the diseases of the *same organs*, governed by the *same laws*, it must follow that any distinction between *it* and physic, is rather nominal than real, and confined mainly to the *practice* in these branches. In presenting the claims of general surgery upon the *dental* surgeon, it will be necessary to bear in mind the proposition set forth and insisted upon in the preceding remarks of this essay, viz. that the numerous individual organs, which make up the human body, although various in structure and function, are all intimately connected, and mutually dependant. Were these different organs of the system so many integral parts, governed by different physiological and pathological laws, and each acting independent of the other, we then might safely confine our investigation to a single part; but considering them, as we must, all merely subordinate parts of one grand machine, and that they all concur, each in its own way, in producing one general result; the life of the individual, we are led to distrust any system, limiting our knowledge to a single organ or suit of organs.

Why does the *general* surgeon pursue general anatomy, and study the laws of the entire system? Is it simply because he is liable to be called upon to treat every part? or rather is it not quite as much to enable himself to treat a single portion with reference to its connections with other parts, and the reciprocal influence between it and all the rest?

Every surgeon, while treating the diseases of a single organ, may be regarded, *pro tem.*, a local surgeon. If, for example, he is treating the diseases of the eye, he acts as oculist; if the ear, as aurist; and if the diseases of the teeth, as dentist. Now, could the general surgeon, simply because he was called to treat a single organ, safely lay aside his knowledge of all the rest?—and if not, what excuse has he, who *by profession* treats a single organ, or suit of organs, for being ignorant of all the remaining ones?

But to be a little more particular, let us inquire whether the *dentist*, whose business it is to treat some of the most delicate portions of the system, parts whose relations to every other organ are the most important, and whose peculiar diseases require a knowledge of every structure and tissue composing the entire frame—whether he is concerned in the investigation of those laws which, according to the present arrangement of medical science, it is the peculiar prerogative of surgery to teach. For example, is the surgeon dentist interested in understanding the subject of irritation? on the one hand its salutary, and on the other its destructive agency?—that irritation in one part is often indicative of disease, or at least of a morbid impression, in a remote one, and hence that to discover the true cause he be able to trace and comprehend fully the laws of transfer? Is he concerned in knowing the different kinds, the comparative effects upon different constitutions, and when seated in different organs and tissues, and, in short, all the modifying circumstances, both intrinsic and extrinsic, its local and constitutional tendencies when protracted, and in solving many other problems connected with this interesting and important subject?

Again, is it of importance that the surgeon dentist have a clear idea of the subject of *inflammation?*—its signs, the different kinds, its stages, the treatment adapted to each stage and kind—its causes, whether, in any given instance, it is more dependant on irritability or debility of the general system, and hence the remedial indications—how varied by structure, and what are its terminations? Should he be familiar with the laws regulating suppuration and ulceration, the treatment of abscesses, and all the various sequelæ of inflammatory action? Than such knowledge, surely, none can be of greater importance to success in the practice of dental surgery. A thorough knowledge of inflammation and irritation is not only of importance in relation to their local effects, but also their termination in general disease. In no case do we see derangement of function more general, nay *universal*, than in *hectic fever*, which is always a remote effect of some local disease, and is uniformly preceded by inflammation and suppuration.

Now, if this local difficulty be situated in the *tibia*, a surgeon must take it in charge, whose knowledge of the system must be beyond all doubt; but if perchance it be located in the mouth, the effect of a score of diseased and dead teeth, attended by extensive disease of the gums and adjacent parts, presenting, as is often the case, several superficial inches of suppurating surface, it may be entrusted to one ignorant of the laws of the human economy, and who is wholly excusable for such ignorance, simply because he is a *dentist!*

May the time soon come, when such logic will meet with that contempt which it so richly merits, and when the dental department of the blacksmith's profession fall into disrepute. That the dentist would not be directly interested in many surgical

*operations,* nor in many of the diseases which the general surgeon is called upon to treat, only as they devolope some law which is of *general* application, will be readily granted; but it may with propriety be remarked, that very few cases of disease can be presented, even where there is no seeming connection, in which the investigation would not disclose to him some law with which he could not be too familiar.

*Theory and Practice of Medicine.*—This department of medical science, is that under which diseases are classed and defined, their symptoms described, their causes investigated, their indications discovered, by which their cure is to be attempted, and the remedies enumerated by which these indications are to be fulfilled. This definition includes, to some extent, general surgery, for, as remarked above, though these two divisions are nominally made, by our present arrangement of medical science, yet this distinction is almost wholly arbitrary. It will be readily perceived, moreover, that to comprehend this branch fully, a knowledge of every other department of medicine is indispensable. But the point in which we are at present interested, is, its applicability to dental surgery. Will, then, the study of theory and practice of medicine augment the resources of the dental surgeon? We answer that it is *capable* of doing so in many very important particulars: by giving more clear and extensive views than he could otherwise obtain of general morbific agents, which are liable to affect every portion of the system, as also *its* susceptibility to be affected by them—by giving direct information upon many diseases which it is the duty of every dental practitioner to understand—and, by bringing out and impressing on his mind a thousand general principles and laws, not only of disease, but of the application and effects of remedies and their indications.

The limited space which this topic can occupy in this dissertation, must prevent very full illustration, and after what has been said of the connections of the various organs of the system, very little time need be spent to prove that any branch, having for its object the investigation of diseases generally, the laws governing them, and their relation to each other, would throw light upon the diseases of any organ or suit of organs, notwithstanding many, or indeed, most of the diseases discussed in works upon this subject, the dentist is never called upon to treat. Among the general morbific agents, the most prominent is impure air. The theory most generally adopted by the medical profession at the present time, in accounting for the various kinds and grades of fever, is, that they have their origin in the impression made upon the nervous system by the different malarious or infectious atmospheres, and that this is effected principally through the agency of respiration. This subject has already been alluded to, but unless we greatly misappreciate its importance, its claims upon the surgeon dentist cannot be too fully impressed.

Did every member of the profession regard it in its true light, as connected with health, far fewer mechanical operations would be performed, where the operator seems but to mimic the agriculturalist, who puts a dead fish upon his hill of corn, that the dissolving elements of the one, may give strength and perfection to the other.

For example, the insertion of pivot teeth upon ulcerated roots, filling cavities with tin, or perhaps mercurial amalgum, in mouths where the fluids are so acrid as to corrode even gold—the insertion of plate teeth over dead and diseased fangs, or the destroying of nerves and plugging them in, lest they should crawl forth and assert their rights. These, and all other similar operations, alike incompatible with a healthy mouth, and the inspiration of pure air, I am persuaded, would be abandoned by the thousands who now perform them, to their own shame and the disgrace of the profession, were they acquainted with the facts which they might learn from any of our modern works on *theory and practice of medicine.* We are indeed of the opinion, that if there were no other subjects discussed in these works, the dental student would be amply repaid for giving them a thorough and careful perusal.

The only remaining subject, or branch of medical science to complete "*the catalogue,*" is obstetrics.

In presenting the claims of this branch to the *dentist,* we may be permitted again to refer to the position, that one of the most important objects which can be attained either by the physician or dentist, is to gain a clear and comprehensive knowledge of the various healthy and morbid sympathies between the various organs of the system—and where, we ask, are these more clearly and fully set forth than in obstetrical works, including as most of them do, infantile diseases. More than a score of diseases, or at least disorders, are described by obstetrical writers, all arising from a *single source* of irritation. We here learn, moreover, that the different organs may not only be affected by direct sympathy, but that a second reflection may occur, endangering often, the most important parts, and all this from *an irritation unnoticed in the organ which is its seat.* Thus, uterine irritation may be first transferred to the stomach or bowels, and thence reflected to the brain, heart or lungs. Hence is accounted for, "pain and giddiness in the head, dimness of sight, convulsions and palsy, palpitation of the heart and peripneumonic complaints," which so frequently accompany pregnancy.

Not only are these direct and reflected sympathetic affections thus established in the different organs, but not unfrequently is the whole habit of body disturbed, while no individual part is perceptibly affected. Thus, a general feverish disposition often occurs, with debility and emaciation, irritability of temper, and in short, all the marks of *general* irritation pervading the entire system.

Were there no other reasons for the dental student pursuing this branch, an ample one would be found in the light which it throws upon these sympathies. Its claims, however, are not merely indirect, but many facts may be learned from such authors, which, to say the least, may save the dentist much mortification.

"Erratic pains in various parts," says Dr. Denman, "especially about the *face, ears and teeth,* so often occur in pregnancy as to be thought certain indications of that state, and which are evidently occasioned by uterine irritation." Not only are these *pains* liable to be mistaken for those arising from dental irritation, but there often occurs positive inflammation of the mouth and gums, which is much more liable to mislead the inexperienced in relation to their real nature and cause. Add to these considerations, the fact, that the dentist has frequently to decide upon the practicability or safety of dental operations during pregnancy, and we have a few of the many requisitions which this branch makes upon the dental practitioner. Although in our discussions of this subject, only a few general particulars have been noticed, yet could no additional incentives be offered, those already enumerated are quite sufficient to induce all, whose chief inquiry is, not after the minimum, homœopathic amount of information, which will possibly answer their end, to give *even this* branch of medical science a careful perusal.

Of infantile diseases, we shall notice only those connected with dentition, and as many of these are treated upon at some length in dental works, our remarks under this head may be brief. At the commencement of our subject, we assumed that he who practices every department of dentistry safely, must be sufficiently acquainted with disease to trace symptoms to their cause, and we may here add, that there are very few instances of disease, in which the connection between cause and effect is not more apparent, and few, hence, where the cause might not be more readily detected from the symptoms presented than those disorders arising from dentition.

Let the *tyro* in dental science be told, that general febrile symptoms are thus produced, that the stomach is disordered, giving rise to vomiting on rising from a recumbent position, or from taking stimulating articles of diet, that a constant disordered state of the bowels is induced, accompanied by costiveness, or diarrhœa, griping and colic pains—that the brain and nerves are often affected, giving rise to sudden startings from sleep, spasmodic affections, convulsions, and acute dropsy of the head—that the whole constitution becomes changed, the temperature of the skin variable—that eruptive diseases, croupy affections, and swellings upon different parts of the body occur, and that all these arise from one and the same cause, simply the irritation of dentition, and it would elicit either his entire incredulity or utmost astonishment, that all these heterogeneous affections should be symptoms of disorder in such a remote and apparently disconnected part. Dr. Eberle remarks, of the time in which this process is going on, that "it is justly regarded as one of the most perilous periods of life." And he adds, "it has been computed that one-tenth at least of all the deaths which occur during childhood may be fairly ascribed to dentition," and, in many instances, "the most alarming consequences result from the irritation of the advancing teeth, before any *signs* of irritation or inflammation are discoverable in the gums."* The principle drawn from the foregoing facts, is one of the greatest practical importance to the dental surgeon. How often we meet with persons, whose whole systems are suffering from extensive dental disease, yet are wholly unconscious of the real origin of their maladies, simply because they had experienced no direct pain or even irritation in their teeth or gums.

This is to be expected on the part of *patients*, but for the *dentist* to be ignorant upon this point, or to fail to give proper advice to such patients, betrays a state of brain which would be greatly benefitted by the lean fare of a penitentiary, and the regular exercise enjoined upon its inmates. While this subject is to be regarded a most important one, its difficulties can but be apparent, as also the culpableness of practising in this department without a thorough acquaintance with the system, and the laws governing both direct and sympathetic disease.

We have now enumerated and set forth some of the claims to the attention of the dental student, the several branches required by law to obtain a "*sheep-skin diploma.*" In relation to several of these branches, we have already had occasion to remark, that they include much which would not *directly* concern the dental student, and which could only be useful to him, as developing and impressing some general principle or law with which he should be familiar.

The student in mathematics, after he had completed the four fundamental rules, might here stop and declare his study properly ended, inasmuch, as every other rule and branch of mathematics are but applications of these four.

But what student, thus ending his course, would be enabled readily to apply his knowledge of this science, to all the various purposes in which its applications are useful? Most clearly no one—and hence the necessity of studying their *applications* in

* Eberle on Children, page 128.

the thousand different forms which make up the great diversity of branches and rules in every department of mathematical science. The whole subject being thus made familiar, the student readily solves the most intricate problems—such, indeed, as would be wholly beyond the comprehension of the beginner. For a similar reason is it useful to study the various branches of medical science. Nor need the student entertain a single fear that he will become too familiar with fundamental facts and principles, by having their *applications*, again and again varied and repeated. There are many in the dental profession who have not only studied in general medical science, but who have practised its principles for a series of years, yet who among us will declare himself unnecessarily familiar with the human system, honorably to discharge the duties devolving upon the dental surgeon? If, after the most careful search, such an one be found, and should he choose as his place of ascension the temple of *Mecca*, I would willingly follow him a pilgrimage there, could I hope that his *mantle* would fall upon *me*. When we hear of such prodigies, they are always afar off, and I have no doubt that distance, uniformly, lends enchantment to the view.

It is, unquestionably, true, that "Sir Astley Cooper, Benjamin Rush, Valentine Mott," and many others, have each of them devoted much more attention to particular portions of medical science than would be profitable as practitioners of dentistry, yet should either of these eminent men "turn dentist," I very much doubt whether he would have his "*vanity inflated*," or his "*efforts paralyzed*," by the profundity of his knowledge of the human system. This is, indeed, far from the tendency of *true* science. It is, in its effects much more like that of *charity*, which "suffereth long and is kind," "envieth not," "vaunteth not itself," and "*is not puffed up*." But while this is true of *real* knowledge, examples are lamentably frequent, where imaginary erudition, has induced its possessor to try the experiment of the *frog* in the fable, and with a similar result. In this light there is quite as much truth as poetry in the saying that,

"A little learning is a dangerous thing."

and the kind of danger, which Pope apprehended, is plain, when he adds, that

> "Shallow draughts intoxicate the brain,
> But drinking largely sobers us again."

This is true, not only in the sense alluded to by the poet, but it is *emphatically* true, also, that "a little learning is a dangerous thing," whenever its possessor assumes the responsibility of practising any department of the healing art.

Though an *ignoramus* may hold up, as a shield, a medical diploma, for such often get them, either *secundum artem*, or otherwise, it may be relied upon, that the "sheep-skin" is neither the *gas* nor *torpedo*, but the malady has a deeper seat in his own specific levity and intrinsic love of quackery. Such an one would make something the basis of a claim to superior excellence, whether he hailed from a medical college, or from Ludgate-Hill. It is not only a matter of serious regret, of extreme mortification, that persons are to be found in every profession, who "would fain steal the livery of the court of heaven to serve the devil in," yet to hold up to ridicule, or damn by faint praise, any institution, whether religious, literary or scientific, because of the *errors*, nay, the flagrant vices of some of its members, would be much like condemning a beautiful field of wheat, simply because it contained an occasional tare.

Though it be admitted that the information which the surgeon dentist should possess, as the basis of his practice, is much encumbered, even by useless material, and that to obtain it from medical works he must act somewhat the part of the miner, yet nothing is more clear, than that in the present state of *dental science*, here a rich vein lies. And it is a comforting reflection, moreover, that in this mine, every laborer, however humble, is a proprietor, and has an inalienable right, to all the rich ore which he may have disencumbered of the mountain masses in which it was buried.

But lest it be inferred that we attach too much importance to a medical diploma, we beg leave to say, that it is entitled to none whatever, unless it be a true certificate, that its possessor has faithfully investigated the several branches required by law to obtain it. As we have already intimated, they are far too easily obtained.

This grows chiefly out of the fact, that they are given by those whose interest it is to grant them. This being a subject which immediately interests us as a society, we shall, in this connection give it a passing notice. Although the law regulating *medical* schools, requires of the student an actual examination, yet this is chiefly conducted by men, in the highest sense interested to confer degrees. This interest is at least two-fold. In the medical institutions of this country the professors are the proprietors of the school—hence the graduation fee (varying from twenty to thirty dollars per head) goes literally into the pockets of the very men, whose business it is, not only to conduct the examination, but to decide upon qualifications.

Again, as these institutions, are regarded prosperous, and, hence, attractive, in proportion to their number of students, and especially graduates, this furnishes another strong inducement to swell the catalogue. To such an extent do these influences prevail in some schools, that their "green-rooms" are literally converted into *ticket-offices.*

This system is radically wrong—for while it divides quackery into licensed and non-licensed, it makes the distinction far too slight between it and science, and often extends a legal protection to those the least deserving.

There can be but little doubt, that it would be far better for community (with this lax administration) were the law to be dispensed with. Could, however, the public be assured that these examinations were a real test of qualification, they would be invaluable. A diploma would then secure confidence to its possessor, and safety to the public. This can only be effected by appointing examiners competent to the task, and wholly disinterested as to the result. Till, at least, some more efficient measures are taken to make diplomas better indices of qualification, we must expect, occasionally, to meet a *wolf* with a *sheep's skin.*

But there is another kind of diploma, where, too often, also, the M. D. would more truly represent the miserable dentist, than doctor of medicine, I refer to what are called honorary degrees.

"Although there may be many high-minded men, who, in preparing themselves diligently and scientifically," even "for dental practice, who *justly* receive these degrees, yet the qualification too frequently required, is an ability and a willingness on the part of the applicant to pay the required fee—for be it understood, that the *fee* is an essential part of the policy, even in granting *honorary diplomas!* In conclusion, we remark, that while we contend, that general medical science should ever constitute one of the chief pillars of the dental art, we boldly assert that no physician, strictly such, is prepared properly to discharge the duties of a dentist. Such, indeed, is their universal ignorance upon this subject that they seem to have regarded the teeth as constituting no part of the human system. Most students, while pursuing their medical course, do, in fact, regard every thing directly pertaining to dentistry as belonging to a distinct branch, in which they have no interest. But no mistake is greater, or productive of more evil, even as applied to him who intends to make physic his sole study and practice. If our views are correct, as set forth in the preceding remarks of this Essay, no physician can be successful in his practice, while he is ignorant of dental diseases, and their relation to the entire frame. But as applied to him who would make a sudden transit from medical to *dental* practice, it is an error which entirely disqualifies him for the latter, even in respect to the recognition and proper treatment of dental diseases, to say nothing of the lack of skill, in those nice and delicate mechanical manipulations, which require years of youthful training to acquire, and that, too, with an original taste which no practice can impart. Whoever, therefore, would reap the full benefit of a medical education, as a dentist, must pursue this study with a direct view to dental practice. For beside the liability of the medical student to neglect as unimportant those facts most useful to the dentist, it is certain, also, that the time actually spent in the study of medicine with a view to obtain a diploma, is never sufficient to enable the student to treasure up, for ready use, but comparatively few of all the facts which are presented in his course, and these would naturally be of that class he deemed most useful. But while every day's observation constrains us to conclude, that the physician is no dentist, it by no means follows that medical science may be neglected with impunity by the dental surgeon. Of the arch which is to stretch across the stream of time and form the basis of his professional eminence, it must, emphatically, constitute the key-stone.

With this brief notice of the several branches of medical science, and their relation to dental practice, I appeal to the good sense and experience of every member of this society, whether there is danger of setting our standard too high. It has been wisely said, that "he who aims at the sun, though he may not reach it, yet his arrow will fly higher than if he aimed at an object on a level with himself."

To *the art of elevating*, it is high time that the attention of every honorable member of the profession was called; "*the art of sinking*," will take care of itself. The history of every quack constitutes an essay upon this subject, and it must be the business of all who would see dentistry occupying its proper rank among the honorable professions, to counteract their wide spread influence. It should not only be his aim to adopt for himself the true standard, but to take all proper means, to disseminate correct views upon this subject.

The fact that dentistry is generally regarded, by the public, as a mere mechanical art, does more towards sustaining quackery, than all other causes combined. It has an effect which *law* can never countervail, and while public opinion is wrong upon this point, no law will be more efficient, than those forbidding the sale of ardent spirits, while public opinion is in favor of this traffic; till this be corrected, in either case, the grunt of the "striped pig" will be heard at every corner of the street—*the fee will be taken at the door, and quackery within dealt out gratis.*

